# Effectiveness of interferential current therapy in patients with knee osteoarthritis: a systematic review and meta-analysis of randomized controlled trials

**DOI:** 10.1038/s41598-022-13478-6

**Published:** 2022-06-11

**Authors:** Hung-Lun Chen, Fu-An Yang, Ting-Hsuan Lee, Tsan-Hon Liou, Reuben Escorpizo, Hung-Chou Chen

**Affiliations:** 1 Department of Primary Care Medicine, Shuang Ho Hospital, Taipei Medical University, New Taipei City, Taiwan; 2 School of Medicine, College of Medicine, Taipei Medical University, Taipei, Taiwan; 3 Department of Family Medicine, Shuang Ho Hospital, Taipei Medical University, New Taipei City, Taiwan; 4 Department of Physical Medicine and Rehabilitation, Shuang Ho Hospital, Taipei Medical University, No. 291 Zhongzheng Road, Zhonghe District, New Taipei City, 235 Taiwan; 5 Department of Physical Medicine and Rehabilitation, School of Medicine, College of Medicine, Taipei Medical University, Taipei, Taiwan; 6 Department of Rehabilitation and Movement Science, College of Nursing and Health Sciences, University of Vermont, Burlington, VT USA; 7 Swiss Paraplegic Research, Nottwil, Switzerland; 8 Center for Evidence-Based Health Care, Shuang Ho Hospital, Taipei Medical University, New Taipei City, Taiwan

**Keywords:** Osteoarthritis, Rehabilitation

## Abstract

We conducted a systematic review and meta-analysis to assess the effectiveness of interferential current therapy (IFC) in patients with knee osteoarthritis. We searched PubMed, Cochrane Library, Embase, ClinicalKey, and Scopus for relevant studies from their date of launch to March 22, 2022. We included randomized controlled trials (RCTs) in which IFC was applied to knee osteoarthritis patients and the outcomes of pain scores or functional scales were assessed. Ten RCTs with 493 patients met the inclusion criteria. Nine RCTs were included in the meta-analysis. The IFC groups exhibited significant improvements relative to the control groups for short-term pain scores (SMD = − 0.64, 95% CI − 1.04 to − 0.25, *P* = 0.001), long-term pain scores (SMD = − 0.36, 95% CI − 0.60 to − 0.11, *P* = 0.005), and short-term Western Ontario and McMaster Universities Osteoarthritis Index scores (SMD = − 0.39, 95% CI − 0.77 to − 0.02, *P* = 0.04). All included studies did not observe any obvious adverse effects of IFC. IFC can be recommended as a treatment for knee osteoarthritis because it improves short- and long-term pain and short-term function. However, large-scale and high-quality RCTs with longer follow-up are required to establish an appropriate standardized treatment.

## Introduction

Knee osteoarthritis is a common degenerative disease, and its prevalence and incidence are increasing; such increases are likely related to the increase in life expectancy, aging population, and overweight rates^1^. Knee osteoarthritis is associated with pain, functional and social impairment, joint stiffness, limited range of motion, and reduced quality of life, and it is one of the major causes of disability worldwide^2^. Local and spreading central sensitization are mechanisms that may contribute to pain in knee osteoarthritis^3^.

The current strategies applied to knee osteoarthritis treatment and rehabilitation are multidisciplinary, and they include surgical and nonsurgical (pharmacological and nonpharmacological) interventions^4^. Commonly used nonpharmacological techniques include exercise, taping, manual therapy, ultrasound, acupuncture, laser therapy, and electrotherapy^5,6^.

Interferential current therapy (IFC) is a highly promising electrotherapy agent for knee osteoarthritis that is currently being developed^5–7^. IFC delivers medium-frequency alternating currents that cross paths to produce interference and generate a low-frequency current known as amplitude-modulated frequency (which is an additional advantage). Amplitude-modulated frequency can penetrate more deeply into tissues and may have an analgesic effect^8^. Wearable IFC devices are being developed because of their user-friendliness^9^.

Previous meta-analyses and reviews have included few trials and reported inconsistent results. A 2009 systematic review^10^ reported inconclusive results regarding the analgesic effects of IFC, whereas more recent meta-analyses have reported IFC as a promising pain relief treatment option; however, all of them included fewer than three trials in the IFC groups^5,6,11^. Therefore, we conducted a systematic review and meta-analysis of a larger number of randomized controlled trials (RCTs) to examine the current body of evidence regarding the effectiveness of IFC (relative to control groups) in adults with knee osteoarthritis.

## Methods

### Study framework, design, and registration

The PICOS (patients, intervention, comparison, outcomes, study design) framework of this study is as follows: A systematic review and meta-analysis of RCTs was conducted (S) to investigate whether patients diagnosed with knee osteoarthritis (P) will show improvement in pain scores or functional scales (O) with the application of IFC (I), when compared to the control groups (C). This study is reported as per the Preferred Reporting Items for Systematic Reviews and Meta-analyses (PRISMA) guidelines^12^. This study was prospectively registered in the international prospective register of systematic reviews (PROSPERO) under registration number CRD42021272873.

### Data sources and retrieval

An online search for relevant articles was conducted by two reviewers independently; the databases used comprised PubMed, Cochrane Library, Embase, ClinicalKey, and Scopus. The following keywords and keyword combinations (relating to the disease and intervention) were used: (interferential OR IFC OR IFT) AND (knee OR osteoarthr* OR arthr* OR OA). The search term (AND (random*)) was added to the aforementioned keywords to identify RCTs when the databases did not provide a filter for searching RCTs. The detailed search strategy is provided in Supplementary Appendix 1. We also manually searched the references in the relevant articles to identify more eligible articles. All databases were searched from their date of launch to March 22, 2022.

### Study selection and eligibility criteria

Two independent reviewers screened these RCTs based on the title, abstract, and full text to identify relevant studies. Only studies that met the inclusion criteria were selected. The opinions of the two reviewers were subsequently compared, and inconsistencies were resolved by a third reviewer through repeated discussions.

We included RCTs in which IFC was applied to patients diagnosed as having knee osteoarthritis and the outcomes of pain scores or functional scales were assessed. RCTs that applied IFC as a co-intervention were also included when the combined intervention was also applied to the control groups, and the effectiveness of IFC could be isolated through a comparison. No language-related restrictions were applied during article selection.

Studies that were protocols, conference papers, and animal studies were excluded. We also excluded RCTs that applied combined treatments, such that the effectiveness of IFC could not be isolated through a comparison with the control groups. We assessed the methodological quality of the studies by using the Physiotherapy Evidence Database (PEDro) scale to exclude trials of poor quality^13^.

### Data items

Two reviewers independently extracted data on the following parameters for the IFC and control groups: number of patients, sex, age, inclusion and exclusion criteria, treatment arm comparisons, follow-up durations, placement and duration of treatment, IFC settings, and appraised outcome measures. Outcome measures assessed immediately after treatment completion were analyzed to determine the short-term effects of IFC, and those assessed during the longest-reported follow-ups (at least 2 months after treatment completion) were analyzed to determine the long-term effects of IFC. The main appraised outcome measures were pain scores and functional scales. Any other outcomes that were reported in more than two RCTs were included in our meta-analysis. Any reported adverse events were recorded.

### Risk-of-bias assessment

The methodological quality of each study was assessed using the Physiotherapy Evidence Database (PEDro) scale, which is a valid measurement tool widely used to evaluate risk of bias^13^. The PEDro scale consists of 11 items that are rated Yes or No (which correspond to 1 and 0 points) depending on whether a criterion is clearly met by a study. However, an item pertaining to external validity was not used in calculations. A total PEDro score of between 0 and 10 was obtained by adding the ratings for the other 10 items, which are as follows: random allocation, concealed allocation, baseline comparability, blinding of participants, blinding of therapists, blinding of assessors, adequate follow-up (more than 85%), intention-to-treat analysis, between-group statistical comparisons, and reporting of point measures and measures of variability. Scores of 0–3, 4–5, 6–8, and 9–10 are considered poor, fair, good, and excellent quality, respectively^14^. Each study was reviewed by the two reviewers and classified as a poor-, fair-, good-, or excellent-quality study.

### Statistical analysis

Data analysis was performed using Review Manager (RevMan) software (Version 5.4.1, The Cochrane Collaboration, London, United Kingdom). The study was performed according to PRISMA guidelines^12^. All relevant data measured using different scales were converted to a single scale for the meta-analysis by using standard mean difference (SMD). Data were pooled using a random-effect model. The precision of effect sizes was reported as 95% confidence intervals (CIs). The heterogeneity of the studies was determined through *I*^2^ tests; *I*^2^ value of 25%, 50%, and 75% indicate low, moderate, and high heterogeneity, respectively^15^. An *I*^2^ value of more than 75% indicates significant heterogeneity, and a sensitivity test is then conducted to verify its effect. Statistical significance was set at *P* < 0.05.

## Results

### Study collection

**Figure 1:**
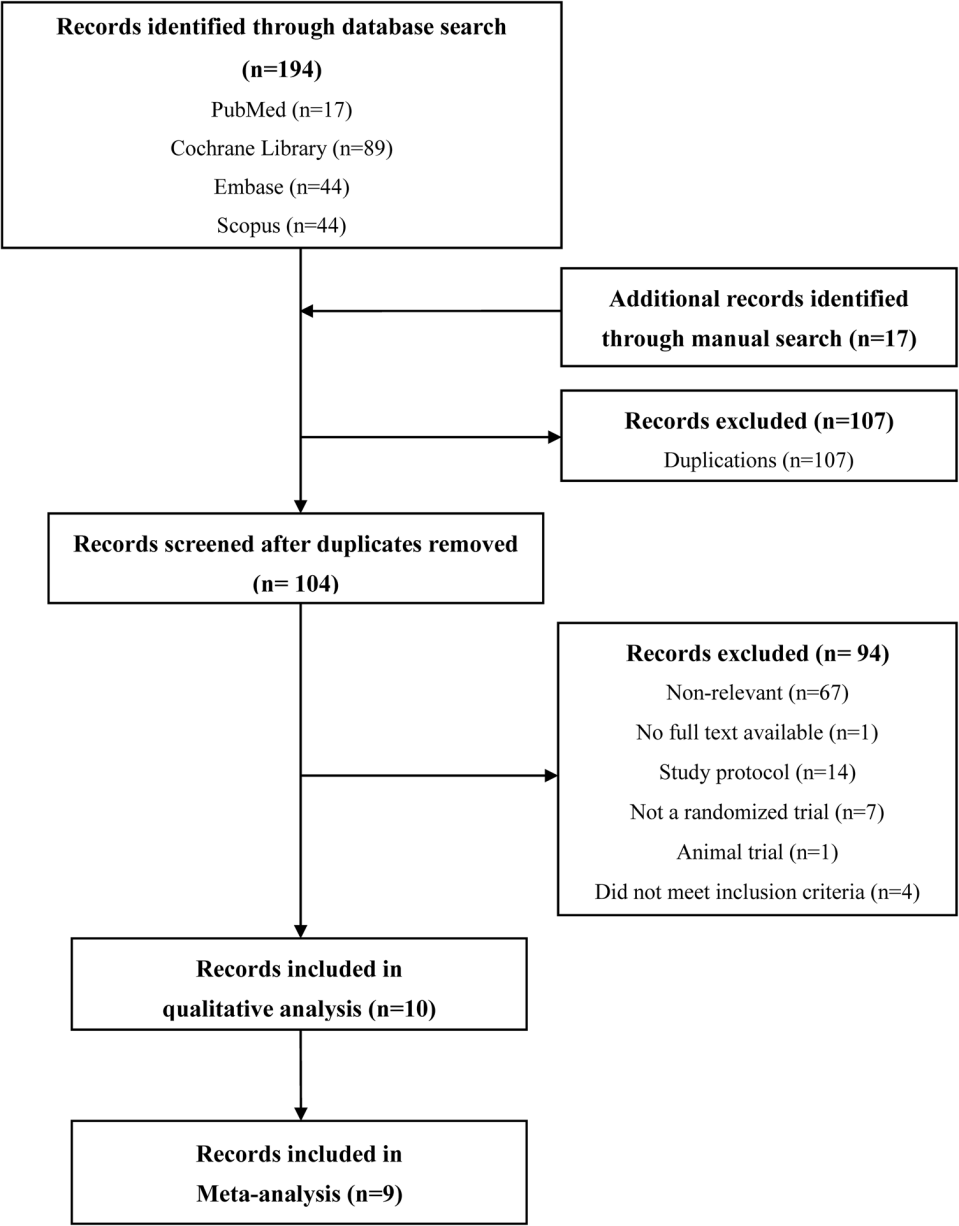
Flow chart for study selection. *n* number.

In total, 194 articles were initially retrieved after the aforementioned search terms were applied. 17 additional articles were identified through manual search. 107 articles were then identified as duplicate publications and were removed. After reviewing the remaining articles’ titles and full texts, 67 articles were identified as irrelevant articles and were excluded. Among the remaining 33 articles, 10 RCTs met the eligibility criteria^16–25^, and nine RCTs^17–25^ were pooled into our meta-analysis; they were all parallel studies. A detailed flowchart of the study selection process is presented in Fig. 1.

### Study characteristics

The 10 selected RCTs were published between 1985 and 2021. From the study arms of each RCTs, a total of 493 patients (262 and 231 patients from the IFC and control groups, respectively) were included in further analysis. All 10 RCTs reported the effectiveness of IFC in reducing the pain or improving the function of patients diagnosed with knee osteoarthritis.

Nine trials^17–25^ reported improvement of pain as a main outcome by using the visual analog scale (VAS) or the numeric rating scale (NRS)^26^. For the assessment of functional capacity, seven trials^18,20–25^ used the Western Ontario and McMaster Universities Osteoarthritis Index (WOMAC), which assesses the domains of pain, joint stiffness, and physical activity^27^.

Three trials assessed mobility function by measuring the time taken to complete walk tests^22,23,25^. Three trials reported stiffness by using the VAS or WOMAC (specifically the stiffness domain)^19,23,24^. The electrical intervention applied by Itoh et al. was labeled as “transcutaneous electrical nerve stimulation,” but the description of this intervention clearly indicated that it was IFC^20^. The main characteristics of these 10 RCTs are summarized in Table 1.

**Table 1:** Summary of the characteristics of the included studies.

Study	N	Selected study arms	Co-intervention	Follow-up	IFC in intervention group		Appraised outcome measure
					Protocol	IFC settings	
Quirk et al.^16^	26	(1) Active IFC + exercises (2) Exercises^‡^	Exercise program (twice/d for 4 wk)	3–6 mo	15 min/d 3 d/wk for 4 wk	Pulse frequency: 0–100 Hz for 10 min, 130 Hz for last 5 min Pulse width and amplitude: N/A Electrodes: Suction electrodes (placement N/A)	Overall clinical score
Adedoyin et al.^17^	30	(1) Active IFC + exercises (2) Sham IFC + exercises^‡^	Dietary advice + Exercise program (twice/wk for 4 wk)	At treatment completion	20 min/d 2 d/wk for 4 wk	Pulse frequency: AMF at 100 Hz for 15 min, 80 Hz for last 5 min Amplitude: Above sensory threshold Electrodes: 2 placed at latero-medial region, 2 placed at antero-posterior region	Pain (VAS)
Adedoyin et al.^18^	31	(1) Active IFC + exercises (2) Exercises^‡^	Exercise program (twice/wk for 4 wk)	At treatment completion	20 min/d 2 d/wk for 4 wk	Pulse frequency: Continuous 80 Hz Amplitude: Above sensory threshold with tingling sensation Electrodes: 2 placed longitudinally at each side of knee	Pain (VAS), function (WOMAC)
Defrin et al.^19^	54	(1) Active IFC (2) Sham IFC^†^	Nil	At treatment completion	20 min/d 3 d/wk for 4 wk	Pulse frequency: Carrier at 4000 Hz and AMF at 30–60 Hz Amplitude: 30% above (noxious) or 30% below (innocuous) sensory threshold; sensation maintained by raising intensity in adjusted group Electrodes: 1 placed at lateral side of knee, 1 placed at medial side of knee	Pain (VAS), stiffness (VAS)
Itoh et al.^20^	24	(1) Active IFC (2) Control^†^ (3) Active IFC + acupuncture (4) Acupuncture^‡^	Control: Topical poultice if required Acupuncture: Six acupoints on affected knee (once/wk for 5 wk)	10 wk	15 min/d 1 d/wk for 5 wk	Pulse frequency: Carrier at 4000 Hz and 4122 Hz, AMF at 122 Hz Amplitude: 2–3 times above sensory threshold Electrodes: A 809-mm^2^ electrode placed at the most tender site, a 5688-mm^2^ electrode placed at its opposite side	Pain (VAS), function (WOMAC)
Dyson^21^	24	(1) Active IFC + exercises (2) Exercises^‡^	Exercise program (twice/wk for 3 wk)	At treatment completion	25 min/d 2 d/wk 3 wk	Pulse frequency: Carrier at 3850 Hz and AMF at 80–120 Hz Electrodes: 4 placed around symptomatic knee	Pain (VAS), function (WOMAC)
Atamaz et al.^22^	66	(1) Active IFC + exercises (2) Sham IFC + exercises^‡^	Exercise program (3 d/wk for 3 wk)	1, 3, and 6 mo	20 min/d 5 d/wk 3 wk	Pulse frequency: Carrier at 4000 Hz and AMF at 100 Hz Amplitude: Tactile sensory threshold Electrodes: 2 placed at knee region	Pain (VAS), function (WOMAC), mobility (15-m walk test)
Gundog et al.^23^	30	(1) Active IFC (2) Sham IFC^†^	Nil	1wk	20 min/d 5 d/wk 3 wk	Pulse frequency: Carrier at 4000 Hz and AMF at 100 Hz Amplitude: Strong but comfortable level Electrodes: 2 placed laterally on the patella	Pain (VAS), function (WOMAC), mobility (15-m walk test), stiffness (WOMAC)
de Paula Gomes et al.^24^	40	(1) Active IFC + exercises (2) Sham IFC + exercises^‡^	Exercise program (3 d/wk for 8 wk)	At treatment completion	40 min/d 3 d/wk 8 wk	Pulse frequency: Carrier at 4000 Hz and AMF at 75 Hz Amplitude: Strong but comfortable level Electrodes: 4 placed around affected knee	Pain (VAS), function (WOMAC), stiffness (WOMAC)
Alqualo-Costa et al.^25^	84	(1) Active IFC + sham PBM (2) Sham IFC + sham PBM^‡^ (3) Active IFC + active PBM (4) Sham IFC + active PBM^‡^	PBM: low-level laser, 27 J per session; average power, 40 mW; cross-sectional area, 0.5 cm^2^	3 and 6 mo	30 min/d 3 d/wk 4 wk	Pulse frequency: Carrier at 4000 Hz and AMF at 50–100 Hz Amplitude: Strong level that is not painful Electrodes: 4 placed with quadripolur configuration to cover area of pain	Pain (VAS), function (WOMAC), mobility (timed up and go test)

*N* number of patients, *IFC* interferential current therapy, *d* day, *wk* week, *mo* month, *min* minute, *Hz* hertz, *N/A* not applicable, *VAS* visual analog scale, *ROM* range of motion, *AMF* amplitude-modulated frequency, *WOMAC* Western Ontario and McMaster Universities Osteoarthritis Index, *PBM* Photobiomodulation.

^†^IFC versus Placebo/Sham.

^‡^IFC + Other therapy versus other therapy.

### Risk-of-bias assessment

The risks of bias of the selected RCTs was assessed using the PEDro scale^13,14^. This assessment was reviewed by two reviewers independently. The studies obtained scores of 4–9 points for overall quality; as per the assessment, six studies^16–18,21–23^ were good-quality studies, two^19,20^ were fair-quality studies, and the two most recent studies^24,25^ were excellent-quality studies.

All the included studies reported adequate baseline comparability, between-group statistical comparisons, point estimates, and variability measures for at least one key outcome. The therapists in all the studies were not blinded. Seven studies clearly stated that assessors were blinded. Three studies reported adequate blinding of patients. Nine studies implemented random allocation. Four studies implemented concealed allocation. Six studies reported follow-up rates of more than 85% for at least one key outcome. Seven studies were analyzed using the intention-to-treat method. The detailed results are summarized in Table 2.

**Table 2:** Summary of methodological quality based on PEDro scale.

Studies included	PEDro scale items										PEDro score	Methodological quality
	1	2	3	4	5	6	7	8	9	10	(0–10)	
Quirk et al.^16^	Y	N	Y	N	N	N	Y	Y	Y	Y	6	Good
Adedoyin et al.^17^	N	N	Y	Y	N	Y	N	Y	Y	Y	6	Good
Adedoyin et al.^18^	Y	N	Y	N	N	Y	Y	N	Y	Y	6	Good
Defrin et al.^19^	Y	N	Y	N	N	N	N	N	Y	Y	4	Fair
Itoh et al.^20^	Y	N	Y	N	N	N	N	N	Y	Y	4	Fair
Dyson^21^	Y	Y	Y	N	N	Y	N	Y	Y	Y	7	Good
Atamaz et al.^22^	Y	Y	Y	N	N	Y	Y	Y	Y	Y	8	Good
Gundog et al.^23^	Y	N	Y	N	N	Y	Y	Y	Y	Y	7	Good
de Paula Gomes et al.^24^	Y	Y	Y	Y	N	Y	Y	Y	Y	Y	9	Excellent
Alqualo-Costa et al.^25^	Y	Y	Y	Y	N	Y	Y	Y	Y	Y	9	Excellent

Items: 1-Random allocation; 2-Concealed allocation; 3-Baseline comparability; 4-Blinded participants; 5-Blinded therapists; 6-Blinded assessors; 7-Adequate follow-up; 8-Intention-to-treat analysis; 9-Between-group comparisons; 10-Point estimates and variability.

Methodological quality: Excellent, 9–10 points; Good, 6–8 points; Fair, 4–5points; Poor, 0–3 points;

Yes (**Y**), 1 point; No (N), 0 point.

### Synthesis of results

#### Pain scores

**Figure 2:**
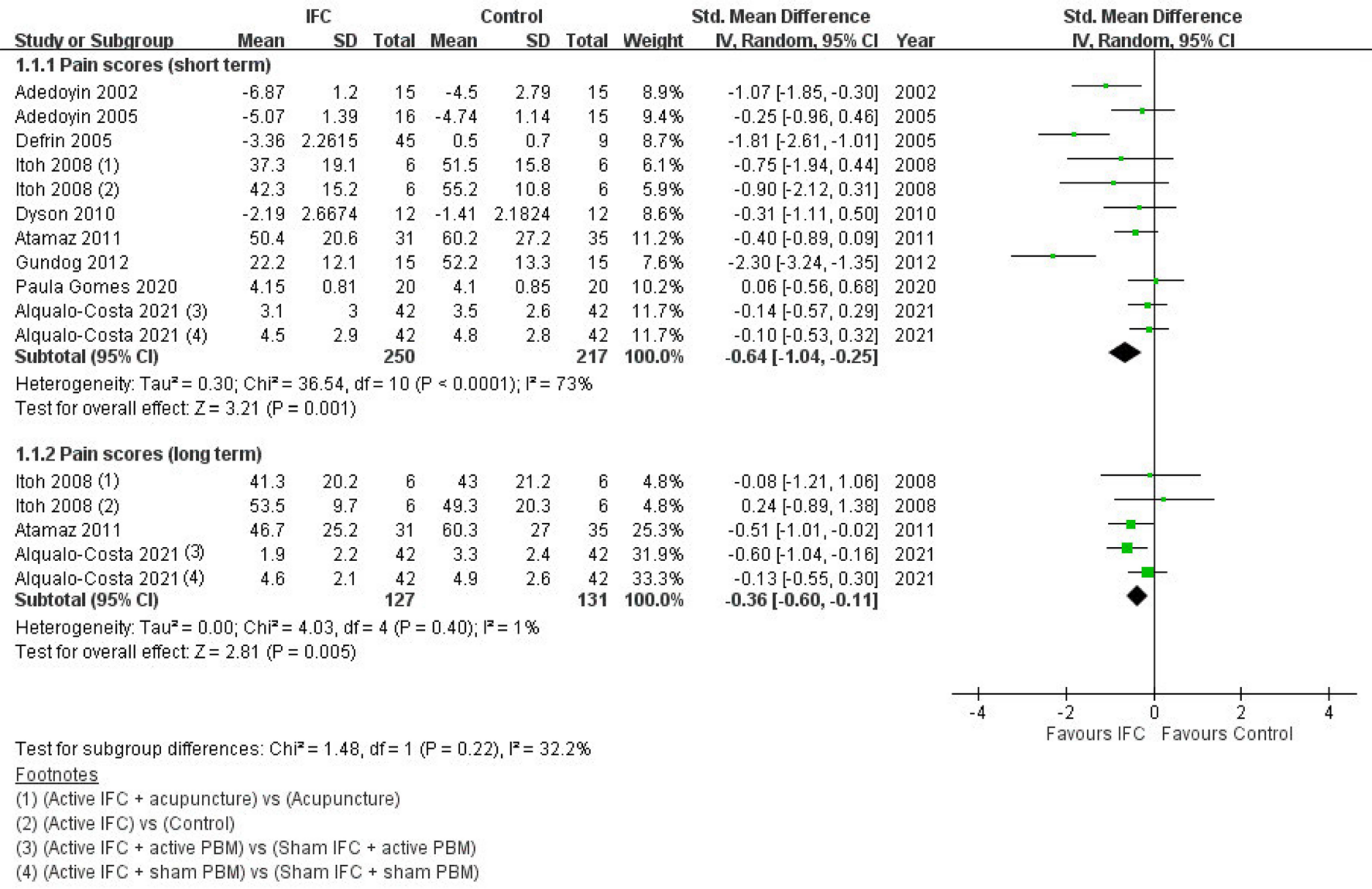
Forest plot of pain scores of IFC and control groups. *IFC* interferential current therapy, *PBM* photobiomodulation, *95% CI* 95% confidence interval.

Pain scores assessed immediately after treatment completion and during follow-up (at least 2 months after treatment completion) were analyzed and labeled as short-term and long-term pain, respectively. Our results indicated that the IFC groups exhibited significant improvement for both short- and long-term pain relative to the control groups. For short-term pain, all nine RCTs were included, and heterogeneity across studies was moderate (SMD = − 0.64; 95% CI − 1.04 to − 0.25; *n* = 467; *I*^2^ = 73%)^17–25^. For long-term pain, three studies were included, and heterogeneity across studies was low (SMD = − 0.36; 95% CI − 0.60 to − 0.11; *n* = 258; *I*^2^ = 1%)^20,22,25^. The forest plot for pain scores is presented in Fig. 2.

#### Functional status

WOMAC scores assessed immediately after treatment completion and during follow-up (at least 2 months after treatment completion) were analyzed and labeled as short- and long-term WOMAC, respectively. Four of the included RCTs only reported the total WOMAC score, whereas the other three only presented the scores for the subscales, from which we selected the data pertaining to the function domain (which accounted for more than 50% of the total WOMAC score) for further analyses^22–24^. The initial meta-analysis of short-term WOMAC included seven RCTs, and the results indicated that the IFC groups achieved significant improvements in WOMAC scores relative to the control groups (SMD = − 0.7; 95% CI − 1.24 to − 0.15; *n* = 383)^18,20–25^. However, the studies exhibited high heterogeneity (*I*^2^ = 83%; *P* = 0.01); therefore, a sensitivity test was conducted by removing data from one of the studies which was a significant outlier, and it reduced the *I*^2^ value to 62%^23^. The results revealed that the significance of meta-analysis was reliable (SMD = − 0.39; 95% CI − 0.77 to − 0.02; *n* = 353; *I*^2^ = 62%).

**Figure 3:**
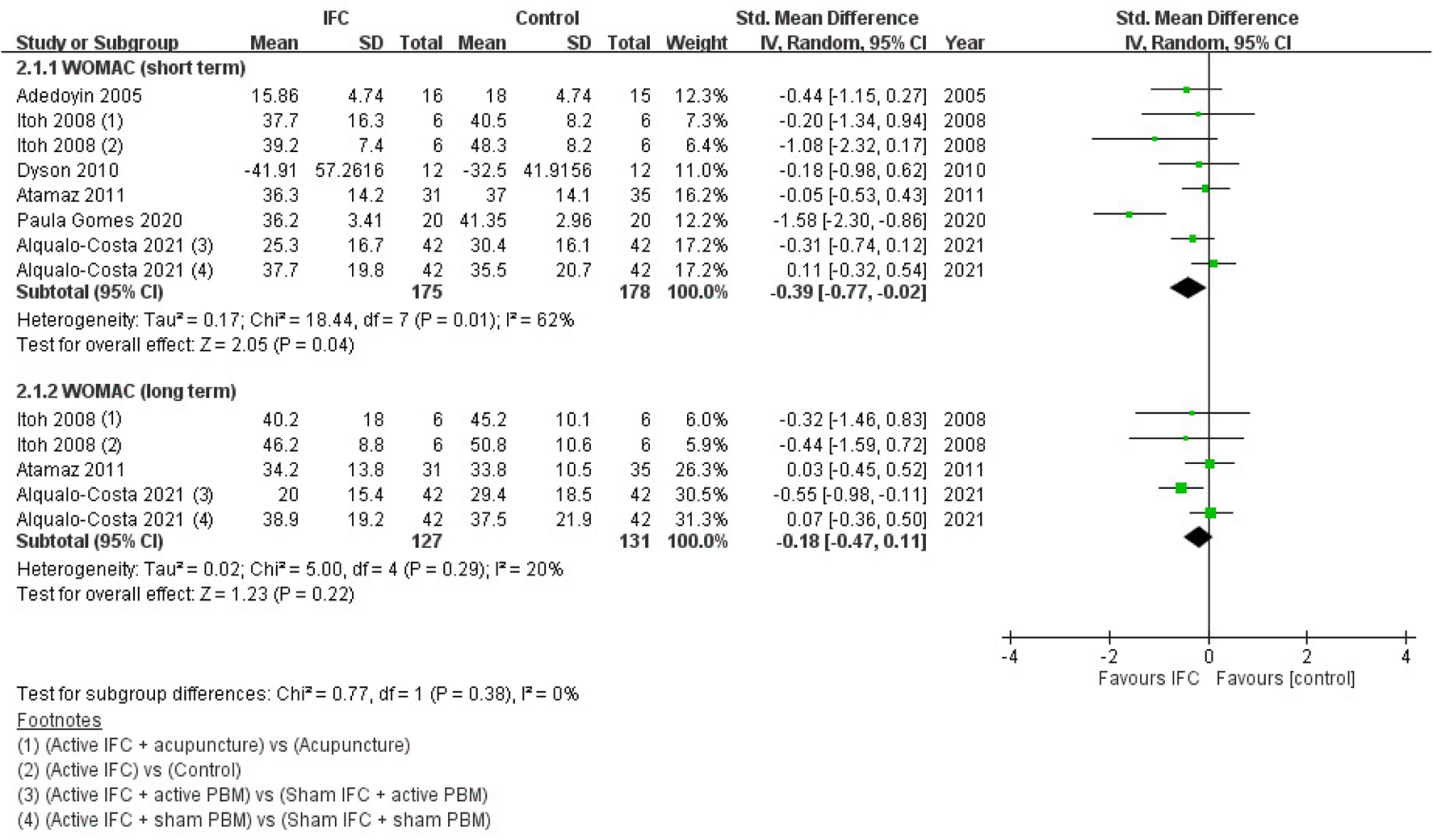
WOMAC scores of IFC and control groups after sensitivity analysis. *WOMAC* Western Ontario and McMaster Universities Osteoarthritis Index, *IFC* interferential current therapy, *PBM* photobiomodulation, *95% CI* 95% confidence interval.

For long-term WOMAC, three studies were included, and they indicated no significant improvements with the implementation of IFC relative to the control groups (SMD = − 0.18; 95% CI − 0.47 to 0.11; *n* = 258; *I*^2^ = 20%)^20,22,25^. The forest plot of WOMAC results after sensitivity analysis is presented in Fig. 3.

#### Mobility status

**Figure 4:**
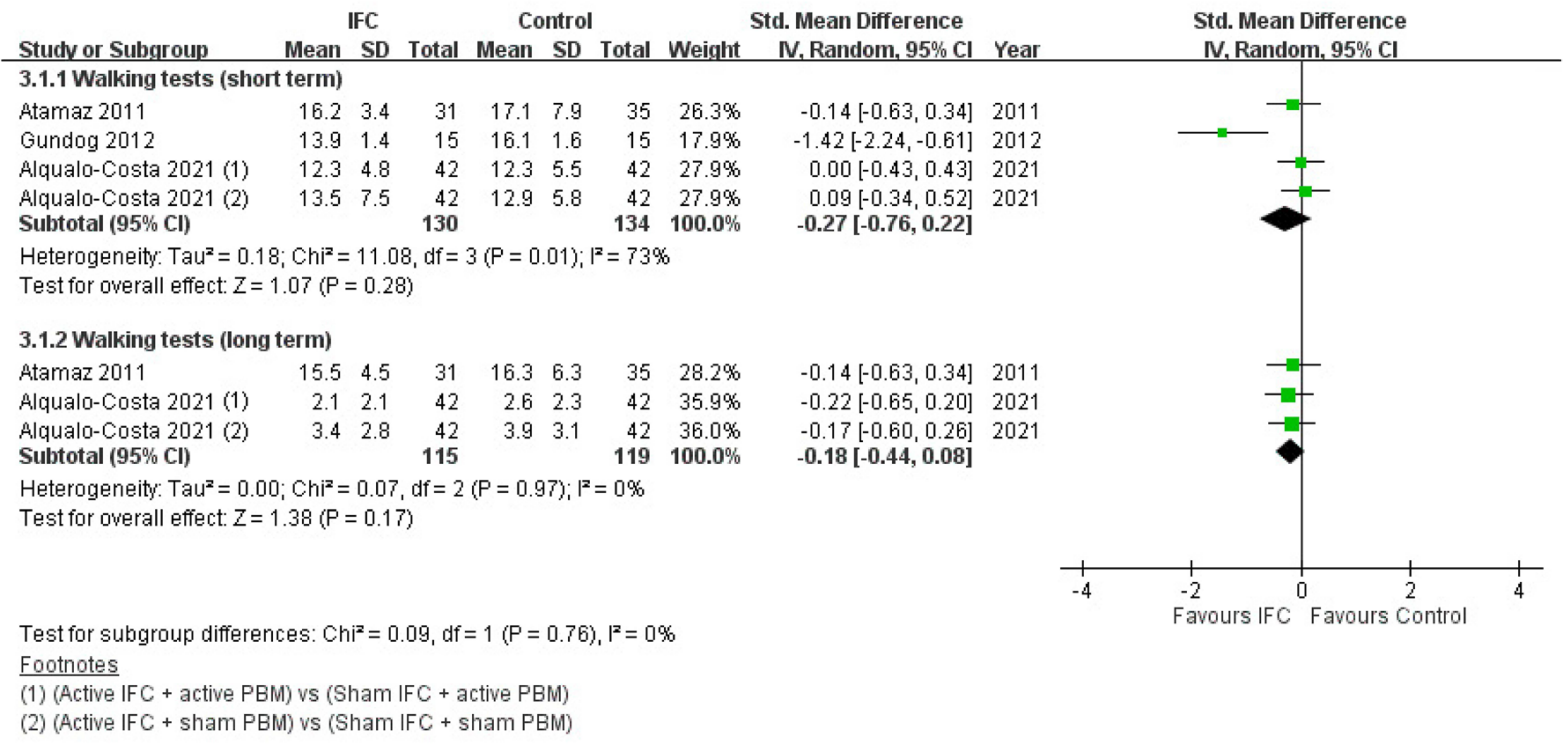
Forest plot of walk test results of IFC and control groups. *IFC* interferential current therapy, *PBM* photobiomodulation, *95% CI* 95% confidence interval.

Mobility status was evaluated by measuring the time taken to complete walk tests, which included the Timed Up and Go test and the 15-m walk test. The results of walk tests conducted immediately after treatment completion and during follow-up (at least 2 months after treatment completion) were analyzed and labeled as short- and long-term walk test results, respectively. Our results indicated no statistically significant differences between the IFC and control groups for both short- and long-term walk test results. For short-term walk test results, three RCTs were included, and heterogeneity across studies was moderate (SMD = − 0.27; 95% CI − 0.76 to 0.22; *n* = 264; *I*^2^ = 73%)^22,23,25^. For long-term walk test results, two studies were included, (one RCT^23^ was not included due to its short follow-up period), and their heterogeneity was low (SMD = − 0.18; 95% CI − 0.44 to 0.08; *n* = 234; *I*^2^ = 0%)^22,25^. The forest plot of walk test results is presented in Fig. 4.

#### Stiffness score

**Figure 5:**
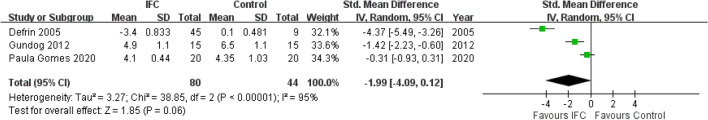
Forest plot of short-term stiffness results of IFC and control groups. *IFC* interferential current therapy, *95% CI* 95% confidence interval.

Three RCTs assessed stiffness using the VAS or WOMAC (specifically the stiffness domain)^19,23,24^. Stiffness scores obtained immediately after treatment were analyzed and labeled as short-term stiffness. None of the RCTs collected long-term follow-up data for stiffness. Our meta-analysis of short-term stiffness scores indicated no significant difference between the IFC and control groups. Heterogeneity across the studies was high (SMD = − 1.99; 95% CI − 4.09 to 0.12; *n* = 124; *I*^2^ = 95%). The forest plot of short-term stiffness scores is presented in Fig. 5*.*

### Studies excluded from meta-analysis

One trial was excluded from the meta-analysis because the functional outcome reported was different from those reported by the other trials^16^. This trial reported a significant improvement in overall clinical scores for both the exercise-only and IFC—exercise groups, but did not verify whether the IFC—exercise intervention was superior to the exercise-only intervention.

### Adverse effects

All included studies did not observe any serious adverse effects of IFC. Good adherence to IFC and low drop-out rates were reported by four studies^22–25^. Only one study observed a deterioration of symptoms in two patients, resulting in these patients dropping out of the study (one patient was in the acupuncture − IFC group, and the other was in the acupuncture control group)^20^. However, the association between symptom deterioration and IFC was unclear.

## Discussion

The main goals of knee osteoarthritis management are to reduce pain and other symptoms and improve functional capacity. IFC is widely used worldwide as a nonpharmacological and nonsurgical intervention; however, there is limited evidence about its effectiveness in knee osteoarthritis management^6,7^. Our findings revealed that immediately after treatment completion, knee osteoarthritis patients who received IFC exhibited significant improvements for both short-term pain and WOMAC scores relative to the control groups. For long-term follow-up (assessed at least 2 months after treatment completion), the patients in the IFC groups also exhibited significant improvements in pain scores relative to the control groups. However, the IFC groups did not exhibit any significant improvements in WOMAC scores over long-term follow-up. The results for short-term stiffness scores, short-term walk tests, and long-term walk tests did not favor IFC.

A 2014 network meta-analysis reviewed relevant literature and reported the significant effects of IFC in improving pain scores at last follow-up^5^; these results are consistent with our findings. Furthermore, the aforementioned meta-analysis indicated that IFC is most likely the best treatment option (among multiple electrical stimulation methods) for pain relief; however, only three of the selected trials directly compared the IFC and control groups, and outcomes other than pain relief were not analyzed^5^. A 2009 systematic review analyzed five IFC-related RCTs, which reported inconclusive results for the effects of IFC on pain relief^10^. A 2019 meta-analysis also reported that IFC is a promising intervention for pain and functional improvement; however, it only included two RCTs that applied IFC; thus, its conclusion should be interpreted with caution^6^.

Our results indicated the positive analgesic effects of IFC in both the short and long term. The timing of follow-up in the selected studies ranged from 10 weeks to 6 months^20,22,25^. The advantage of IFC over other electrical treatment options is the generation of amplitude-modulated frequency, which enables deeper penetration into tissues and has been suggested as a main analgesic component^8^. Studies have suggested that chronic osteoarthritis pain is related to central sensitization, and electrical nerve stimulation interventions such as IFC may target central sensitization by modulating pain and desensitizing the central nervous system, resulting in a long-term analgesic effect^25,28^.

In the present study, functional status was primarily assessed using the WOMAC scale, and significant improvements with the implementation of IFC were observed in short-term assessment but not in long-term follow-up. We also discovered that the results for walk tests (short- and long-term) and stiffness scores (short-term) did not favor IFC use over control treatment. The WOMAC scale evaluates activities of daily living, functional mobility, gait, general health, and quality of life in knee osteoarthritis patients^27^. It consists of 24 questions that can be divided into the subscales of pain, physical function, and stiffness. The outlier data of one study were removed in sensitivity testing because we observed high heterogeneity, which could be attributed to the study’s inadequate blinding of participants and its small sample size^23^. The significance of our meta-analysis findings persisted after sensitivity testing, indicating that the outcomes were reliable.

A consistent treatment protocol has not yet been established for IFC application, and the RCTs included in our study used varying IFC settings. The RCTs applied a carrier frequency of 3850–4000 Hz and an amplitude-modulated frequency of 30–180 Hz (most RCTs applied an amplitude-modulated frequency of 80–100 Hz). Furthermore, most of the RCTs emphasized that the amplitude of IFC should be above the sensory threshold, which was described as a “strong but comfortable sensation.” Gundog et al. applied three groups of different IFC amplitude-modulated frequencies, namely 40 Hz, 100 Hz, and 180 Hz^23^. The data pertaining to the 100-Hz group were selected for our meta-analysis to reduce heterogeneity with other studies, whereas the data relating to the other two groups were not included to avoid repeating comparisons of the control groups. Gundog et al. reported that varying the amplitude-modulated frequencies did not change the results, and effective pain relief was observed for all frequencies^23^. Furthermore, experimental studies have challenged the analgesic effect of amplitude-modulated frequency, in which they investigated whether varying the carrier frequency or amplitude of IFC plays a more important role in influencing pain relief outcomes^29,30^. A 2022 systematic review even reported that most IFC parameters do not seem to influence its analgesic effects^31^. While Defrin et al. reported that IFC interventions in which an amplitude that was 30% above the pain threshold resulted in better analgesic effects (relative to interventions in which the amplitude was 30% below the pain threshold); however, the presence of the placebo effect was also suggested by the study^19^. The other included RCTs did not specify IFC amplitude settings; thus, further research on this topic is required.

The PEDro scale was applied to evaluate the risks of bias of the selected RCTs^13,14^. Most studies implemented random allocation^16,18–25^; however, one study alternately assigned enrolled patients to intervention groups^17^; this randomization process increased the likelihood of introduced bias. Furthermore, although six studies reported the use of sham devices, three of them described the sham IFC machines as being in a “switched-off” state during intervention administration^19,22,23^; thus, the patients participating in these studies could have been inadequately blinded to the intervention; in the other three studies, the sham devices were covered with a towel or their lights were kept on^17,24,25^; thus, these studies were considered to have implemented adequate participant blinding. Due to the nature of physical therapy implementation, the blinding of therapists was not achieved for all of the selected studies^16–25^. Although the overall quality of the studies selected for our meta-analysis were determined to be of fair to excellent quality, the aforementioned risks of bias should still be considered.

Although previous IFC studies have reported adverse effects such as burns and vasovagal reactions, no adverse effects of similar severity were reported by the studies selected in the present research^32,33^. With respect to the RCT in which a patient from the acupuncture—IFC group dropped out, the association between IFC and symptom deterioration was unclear, and no further follow-up of this patient was reported^20^. Thus, IFC can still be generally considered as a safe and well-tolerated intervention^10^.

The present study has several strengths. First, compared with previous studies, the present study included a larger number of RCTs to examine the current body of evidence supporting the effectiveness of IFC for knee osteoarthritis. Second, without imposing language restrictions, comprehensive searches of multiple key databases were conducted to identify appropriate RCTs. Third, our research can serve as a foundation for future studies, given that multiple clinical trials are currently being conducted in this field. Fourth, the data and quality of the selected studies were extracted and assessed by at least two reviewers through group consensus.

However, the present study has several limitations. First, the IFC devices, IFC parameter settings, and treatment protocols used by the included studies were inconsistent, which may result in moderate-to-high heterogeneity for some results. Further research is required to establish an appropriate standardized treatment. Second, some studies did not implement adequate blinding of therapists and participants, resulting in risks of bias that may have affected the results of the present study. Third, only three of the included RCTs had follow-up durations longer than 2 months^20,22,25^. Fourth, five of the included RCTs reported only the outcome measures assessed immediately after treatment completion, thereby limiting the applicability of the long-term results^17–19,21,24^. Thus, further large-scale and high-quality RCTs with longer follow-up are required to overcome these limitations.

In conclusion, our systematic review and meta-analysis of RCTs assessed the current body of evidence regarding the effectiveness of IFC in adults with knee osteoarthritis. Our analyses indicate that the application of IFC alleviated both short- and long-term knee pain and improved short-term function (as per the WOMAC scale results). The included RCTs did not report any obvious adverse effects. Therefore, IFC can be recommended as treatment for knee osteoarthritis patients. However, high heterogeneity among the IFC parameters and treatment protocols were noted, and trials that directly compared IFC versus other therapy were not included in our study; hence, more studies are required to establish an appropriate standardized treatment.

## Supplementary Information

Supplementary Information.
