# Supplementary material for: Effectiveness of interferential current therapy in patients with knee osteoarthritis: a systematic review and meta-analysis of randomized controlled trials
Source: Sci Rep. 2022 Jun 11;12:9694. doi: 10.1038/s41598-022-13478-6 (PMC9188606; doi:10.1038/s41598-022-13478-6)
Supplement: Supplementary file 1 — Supplementary Information. [file 41598_2022_13478_MOESM1_ESM.docx]

**Title**

Effectiveness of Interferential Current Therapy in Patients with Knee Osteoarthritis: A Systematic Review and Meta-Analysis of Randomized Controlled Trials

**Authors and affiliations**

1^st^ author: Hung-Lun Chen, MD ^1^ (e-mail: mauricechen1209@gmail.com),

2^nd^ author: Fu-An Yang, MD ^2^ (email: yg758312@gmail.com),

3^rd^ author: Ting-Hsuan Lee, MD ^3^ (email: 19190@s.tmu.edu.tw),

4^th^ author: Tsan-Hon Liou, MD, PhD ^4,5^ (email: peter_liou@s.tmu.edu.tw),

5^th^ author: Reuben Escorpizo, DPT ^6,7^ (email: escorpizo.reuben@gmail.com),

6^th^ & *corresponding author: Hung-Chou Chen, MD^4,5,8^ (email: 10462@s.tmu.edu.tw).

^1^Department of Primary Care Medicine, Shuang Ho Hospital, Taipei Medical University, New Taipei City, Taiwan

^2^School of Medicine, College of Medicine, Taipei Medical University, Taipei, Taiwan

^3^Department of Family Medicine, Shuang Ho Hospital, Taipei Medical University, New Taipei City, Taiwan

^4^Department of Physical Medicine and Rehabilitation, Shuang Ho Hospital, Taipei Medical University, New Taipei City, Taiwan

^5^Department of Physical Medicine and Rehabilitation, School of Medicine, College of Medicine, Taipei Medical University, Taipei, Taiwan

^6^Department of Rehabilitation and Movement Science, University of Vermont, College of Nursing and Health Sciences, Burlington, VT, USA

^7^Swiss Paraplegic Research, Nottwil, Switzerland

^8^Center for Evidence-Based Health Care, Shuang Ho Hospital, Taipei Medical University, New Taipei City, Taiwan

**Corresponding author**

Hung-Chou Chen, MD

Department of Physical Medicine and Rehabilitation, Shuang Ho Hospital, Taipei Medical University, No. 291 Zhongzheng Road, Zhonghe District, New Taipei City 235, Taiwan

Tel: +886-2- 22490088 ext. 1603

Fax: +886-2-22480577

E-mail: 10462@s.tmu.edu.tw

**Supplementary Appendix 1: Search strategies for the databases**

| **Database: PubMed** | |
| --- | --- |
| Search# | Keyword(s) |
| #1 | "interferential"[All Fields] |
| #2 | "IFC"[All Fields] |
| #3 | "IFT"[All Fields] |
| #4 | #1 OR #2 OR #3 |
| #5 | "knee"[All Fields] |
| #6 | "joint"[All Fields] |
| #7 | #5 AND #6 |
| #8 | "knee"[MeSH Terms] |
| #9 | "knee"[All Fields] |
| #10 | "knee joint"[MeSH Terms] |
| #11 | "knee joint"[All Fields] |
| #12 | "osteoarthr*"[All Fields] |
| #13 | "arthr*"[All Fields] |
| #14 | "OA"[All Fields] |
| #15 | #7 OR #8 OR #9 OR #10 OR #11 OR #12 OR #13 OR #14 |
| #16 | randomizedcontrolledtrial[Filter] |
| #17 | #4 AND #15 AND #16 |

| **Database: Cochrane Library** | |
| --- | --- |
| Search # | Keyword(s) |
| #1 | "interferential" |
| #2 | "IFC" |
| #3 | "IFT" |
| #4 | #1 OR #2 OR #3 |
| #5 | "knee" |
| #6 | "osteoarthr*" |
| #7 | "arthr*" |
| #8 | "OA" |
| #9 | #5 OR #6 OR #7 OR #8 |
| #10 | trial[Filter] |
| #11 | #4 AND #9 AND #10 |

| **Database: Embase** | |
| --- | --- |
| Search # | Keyword(s) |
| #1 | "interferential" |
| #2 | "IFC" |
| #3 | "IFT" |
| #4 | #1 OR #2 OR #3 |
| #5 | 'knee'/exp |
| #6 | "knee" |
| #7 | "osteoarthr*" |
| #8 | "arthr*" |
| #9 | "OA" |
| #10 | #5 OR #6 OR #7 OR #8 OR #9 |
| #11 | [randomized controlled trial]/lim |
| #12 | #4 AND #10 AND #11 |

| **Database: ClinicalKey** | |
| --- | --- |
| Search # | Keyword(s) |
| #1 | "interferential" |
| #2 | "IFC" |
| #3 | "IFT" |
| #4 | #1 OR #2 OR #3 |
| #5 | "knee" |
| #6 | "osteoarthr*" |
| #7 | "arthr*" |
| #8 | "OA" |
| #9 | #5 OR #6 OR #7 OR #8 |
| #10 | Clinical Trials[Filter] |
| #11 | #4 AND #9 AND #10 |

| **Database: Scopus** | |
| --- | --- |
| Search # | Keyword(s) |
| #1 | "interferential" |
| #2 | "IFC" |
| #3 | "IFT" |
| #4 | #1 OR #2 OR #3 |
| #5 | "knee" |
| #6 | "osteoarthr*" |
| #7 | "arthr*" |
| #8 | "OA" |
| #9 | #5 OR #6 OR #7 OR #8 |
| #10 | "random*" |
| #11 | #4 AND #9 AND #10 |
| #12 | TITLE-ABS-KEY (#11) |
